# Green synthesis of sulfur nanoparticles and evaluation of their catalytic detoxification of hexavalent chromium in water

**DOI:** 10.1039/c8ra07845a

**Published:** 2018-10-25

**Authors:** R. M. Tripathi, R. Pragadeeshwara Rao, Takuya Tsuzuki

**Affiliations:** Amity Institute of Nanotechnology, Amity University Noida Uttar Pradesh 201313 India; Research School of Engineering, College of Engineering and Computer Science, Australian National University Canberra ACT 2601 Australia takuya.tsuzuki@anu.edu.au

## Abstract

Chromium contamination in the aquatic environment is an urgent and serious issue due to its mutagenic and carcinogenic effects against living organisms. The present study demonstrates the capability of biogenic sulfur nanoparticles (SNPs) for the reduction of hexavalent chromium into a less toxic state. A green approach was adapted for the synthesis of SNPs using *F. benghalensis* leaf extract which acts as a reducing and capping agent. The biosynthesized SNPs were characterized by UV-Vis spectroscopy, X-ray diffraction (XRD), Fourier transform infrared spectroscopy (FTIR), atomic force microscopy (AFM), scanning electron microscopy (SEM), high-resolution transmission electron microscopy (HR-TEM) and energy dispersive X-ray spectroscopy (EDX). TEM micrographs revealed that the zero-valent sulfur nanoparticles were in the range of 2–15 nm and the average size of 5.1 nm. The conversion rate of Cr(vi) into Cr(iii) in the presence of SNPs was 88.7% in 80 min. The optimum concentration ratio between SNPs and formic acid was 10 ppm : 480 mM.

## Abstract 2

Biosynthesized sulphur nanoparticles showed high efficiency in the reduction of Cr(vi) even at a small catalyst/Cr(vi) ratio.

## Graphical abstract

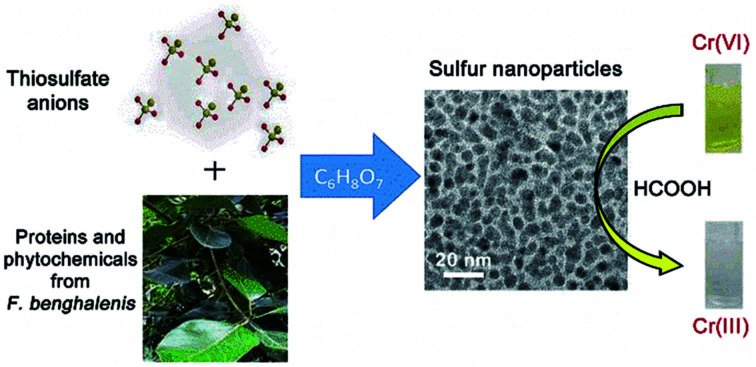

## Introduction

Due to the rapid growth in population and industrialization, increased quantities of toxic pollutants such as heavy metals and synthetic organic matters are being released into the aquatic system. Among these toxicants, chromium is one of the most toxic pollutants.^1^ Chromium is released from the effluents of many industries including leather tanning, paint formulation, wood preservatives, steel fabrication, and metal finishing. The toxicity of chromium depends on its oxidation state as it ranges from +6 to −2. The most stable forms found in the environment are hexavalent chromium Cr(vi), and trivalent chromium Cr(iii). Cr(vi) is associated with oxygen to form chromate CrO_4_^2−^ or dichromate Cr_2_O_7_^2−^ which are highly soluble in water.^2^ Intracellular processes reduce these strong oxidizing agents into Cr(v) which causes damage to nucleic acid and other components of cells to induce mutagenic and carcinogenic effects.^3^

The World Health Organization (WHO) stated in their reports that the Cr(vi) level in drinking water must not exceed 0.05 mg l^−1^.^4^ Cr(iii) is 500 fold less toxic than Cr(vi) towards living organisms.^5,6^ In fact, a trace amount of Cr(iii) present in living organisms acts as an essential nutrient.^7^ Therefore, it is best to consider the reduction of Cr(vi) into Cr(iii) as an effective solution for Cr(vi)-alleviation in drinking water. The conventional methods of Cr(vi) removal such as coagulation, metal adsorption and reverse osmosis require high maintenance and installation costs that can be afforded only in large scale applications.^8,9^ In comparison, catalytic treatments are a low cost, environmentally friendly, flexible in design and commercially feasible technique to eliminate pollutants.^10^

Nanomaterials are excellent catalysts due to their high surface areas.^11–13^ In the past, various nanomaterials have been explored for the catalytic and photocatalytic reduction of Cr(vi) to Cr(iii), including minerals containing Fe(ii), Fe/Ni bimetallic nanoparticles, sulfides, ZnO nanorods, and CdS nanoparticles.^14–16^ However, it is best to avoid the use of toxic materials such as CdS.^17^ Many photocatalysts such as TiO_2_ also have limitations as they work only under UV light. As such, further development of new nanomaterials for the catalytic reduction of Cr(vi) to Cr(iii) is required.

Sulfur in the elemental form has proven to be an effective agent for the conversion of Cr(vi) to Cr(iii).^18^ However, to date, only one study has been published regarding nano-scale elemental sulfur being involved in the reduction of Cr(vi).^19^ In their study, nano-scale elemental sulfur was formed only as a by-product during the reaction between Cr(vi) and H_2_S. In addition, the catalytic reduction was possible only under an anaerobic condition; the presence of oxygen compromised the catalytic efficiency. To the best of our knowledge, nano-scale zero-valent sulfur particles has never been studied as a major catalytic component for the reduction of Cr(vi). In our study, biosynthesized zero-valent sulfur nanoparticles showed excellent catalysis even under aerobic conditions, which is a significant improvement over the previous work and is critical in the practical water-treatment applications.

In the past, only a few reports have appeared on the synthesis of zero-valent sulfur nanoparticles, despite their important applications in energy storage and pharmaceutical products.^20–22^ Recently, the preparation of monoclinic sulfur nanoparticles *via* a microemulsion method was reported.^23^ However, this method releases hazardous H_2_S gas during the synthesis. In order to consider the life-cycle impact of nanotechnology, it is important for nanomaterials to be produced in a sustainable and environmentally friendly manner. For the green synthesis of various nanomaterials, the use of microbes^24,25^ and plant extract^26^ has been reported. The use of plant extract is more advantageous than microbial-mediated processes as it generally requires a shorter reaction time. The phenolic and other chemical compounds within plant extract facilitate the formation of nanomaterials as reducing and capping agents.^27^

In the present study, orthorhombic sulfur nanoparticles (SNPs) were prepared through an environmental friendly process using *F. bengalensis* leaf extract and the catalytic application of SNPs was investigated for the reduction of Cr(vi) to Cr(iii). *F. bengalensis* leaves were selected due to their proven activity as an efficient biomaterial for the synthesis of nanoparticles and ease of availability.^28^ To the best of the authors' knowledge, this is the first time that sulfur nanoparticles with a narrow size range from 2–15 nm were produced using plant extract. The catalytic activity of SNPs was studied in the presence of formic acid, as formic acid undergoes a catalytic transformation to liberate H_2_ and CO_2_ during the dehydrogenation process^29^ and the liberated H_2_ is adsorbed on the surface of the SNPs to pertain to the reduction of Cr(vi) to Cr(iii) through an H_2_ transfer pathway.^30^

## Experimental

### Materials

*F. bengalensis* leaves were procured at the Amity University campus, Noida, India. Sodium thiosulfate pentahydrate (Na_2_S_2_O_3_·5H_2_O, 99.5%), citric acid (C_6_H_8_O_7_), potassium dichromate (K_2_Cr_2_O_7_), formic acid (HCOOH, 85%), sulfuric acid (H_2_SO_4_), hydrochloric acid (HCl), nitric acid (HNO_3_) and sodium hydroxide (NaOH) were purchased from Thermo Fischer Scientific India Pvt Ltd. All chemicals were used as received. A water purification system (Milli-Q Water Purification System) was utilized to obtain deionized water.

### Preparation of leaf extract

*F. bengalensis* leaves were washed thoroughly with tap water and then with deionized water until all the unwanted visible dirt particles were removed. Subsequently, the leaves were allowed to dry at room temperature to remove surface moisture. The dried leaves were chopped into fine pieces and 20 g of them were dispersed into 100 ml deionized water in a 200 ml Erlenmeyer flask. The mixture was boiled at 100 °C for 2 h and filtered using muslin. The filtrate solution was used as leaf extract.

### Synthesis of sulfur nanoparticles (SNPs)

Before the synthesis of SNPs, all the glassware were cleaned using an *aqua regia* solution (3 : 1-nitric acid and hydrochloric acid) to remove the potential nucleation sites on the surface of the glassware. First, sodium thiosulfate pentahydrate (0.078 M) was dissolved in 50 ml deionized water until complete liquefaction. Then, 20 ml of *F. benghalensis* leaf extract was added to the above solution. After 5 min of continuous stirring, 25 ml of 20% citric acid (C_6_H_8_O_7_) was added to the preceding reaction mixture. After the mixture was kept for 1 h at continues stirring, a precipitation was observed. The precipitate was collected after washing with deionized water through centrifugation.

### Characterization of sulfur nanoparticles

UV-Vis spectroscopy (UV-1601 PC, Shimadzu, Japan) was used for the preliminary characterization of as-synthesized SNPs with the scanning range of 200–700 nm. Powder X-ray Diffraction (XRD) patterns were obtained using an X-ray Diffractometer (X'Pert PRO, PANanalytical, Netherland) with CuK_α_ radiation (*λ* = 1.5417 Å) at 40 keV in the range of 20° ≤ 2*θ* ≤ 80°. The QualX and PCPDFWIN software were used to calculate the lattice parameters. The size and morphology of SNPs were characterized by atomic force microscopy (AFM) using a Solver Nano system (NT-MDT, Russia) and the data was analyzed using the Gwyddion software. The AFM samples were prepared by drop-coating the colloidal solution of SNPs onto a cover glass and the analysis was performed in tapping mode using a commercial silicon tip cantilever.

The structural characteristics of biosynthesized SNPs were studied by scanning electron microscopy (SEM, Zeiss, EVO 18, Germany). The SEM specimen was prepared by first drop-coating SNFs on a mica film followed by sputter coating of gold, then the mica film was transferred on to a sample holder made of carbon. The elemental composition of SNPs was identified by energy dispersive X-ray spectroscopy (EDX).

Transmission electron microscopy (TEM, Philips CM-10, Netherlands) observations were made at an accelerating voltage of 300 kV. Particle size distributions were characterized from the TEM images using the ImageJ software.

The biomolecules present on the SNPs were analyzed by Fourier transform infrared spectroscopy (FTIR, 1750, Perkin-Elmer, USA). For FTIR measurements, the colloidal suspension of SNPs was centrifuged at 3000 rpm for 15 min and the pellet was washed four times to remove the impurities. The spectra were recorded in the range of 600–4000 cm^−1^.

### Conversion of Cr(vi) into Cr(iii)

The catalytic activity of biosynthesized SNPs was evaluated for the reduction of Cr(vi) to Cr(iii) under ambient conditions. The catalytic performance of synthesized SNPs was monitored by UV-Vis spectroscopy in the presence of formic acid (HCOOH) as a reducing co-agent. A stock solution of Cr(vi) was prepared from potassium dichromate (K_2_Cr_2_O_7_). A reaction mixture containing 480 mM of 85% formic acid and 20 ml of 200 ppm Cr(vi) solutions was taken in a 50 ml Erlenmeyer flask under constant stirring. Subsequently, 10 ppm SNPs was added to the above reaction mixture to reduce the Cr(vi). In order to evaluate the effect of the presence of SNPs on the reduction of Cr(vi) to Cr(iii), another reaction mixture was also prepared in the same way as for the above solution but without SNPs. The pH of the reaction mixture was adjusted to be 1.25 during the reduction of Cr(vi) to Cr(iii) with H_2_SO_4_. Elemental sulfur does not react with non-oxidizing acids at this pH.^31^ This pH value was chosen following the report of Mohapatra *et al.*^32^ where the conversion rate of Cr(vi) to Cr(iii) was the highest in the pH range from 1 to 2 and increasing pH from 2 to 8 gradually decreased the conversion rate.^32^ The reason for this pH-effect is that, at pH < 2, Cr(vi) forms HCrO_4_^−^ which has higher reactivity than the other forms of Cr(vi) (CrO_4_^−^ and Cr_2_O_7_^2−^) that occur at pH > 2.^32,33^

UV-Vis spectroscopy was used to monitor the catalytic reduction of Cr(vi) by taking 1 ml of the sample from the reaction mixture at particular time intervals. The characteristic absorbance of Cr(vi) at *λ* = 350 nm was used as the reference for the catalytic conversion of Cr(vi) into Cr(iii). The percentage conversion of Cr(vi) into Cr(iii) was calculated using the following equation;

1 % conversion = (1 − *C*/*C*_0_) × 100,

where *C* is the concentration at time *t* and *C*_0_ is the initial concentration.

The colorimetric confirmation of the presence of Cr(iii) was performed by adding an excess amount of sodium hydroxide into the completely reduced Cr(vi) solution. The color change from clear into green indicates the presence of hexahydroxochromate (III), which in turn indicates the presence of Cr(iii).^34^

The effects of catalyst concentration were studied using aqueous solutions containing water (10.0 ml), Cr(vi) (200 ppm), HCOOH (480 mM) at different SNPs concentrations ranging from 1, 5, 10 and 15 ppm. The effects of HCOOH concentration was studied using aqueous solutions containing water (10.0 ml), Cr(vi) (200 ppm), SNPs (10 ppm) at different HCOOH concentrations ranging from 0, 260, 370, 480 and 590 mM.

## Results & discussion

### Sulfur nanoparticles

**Fig. 1:**
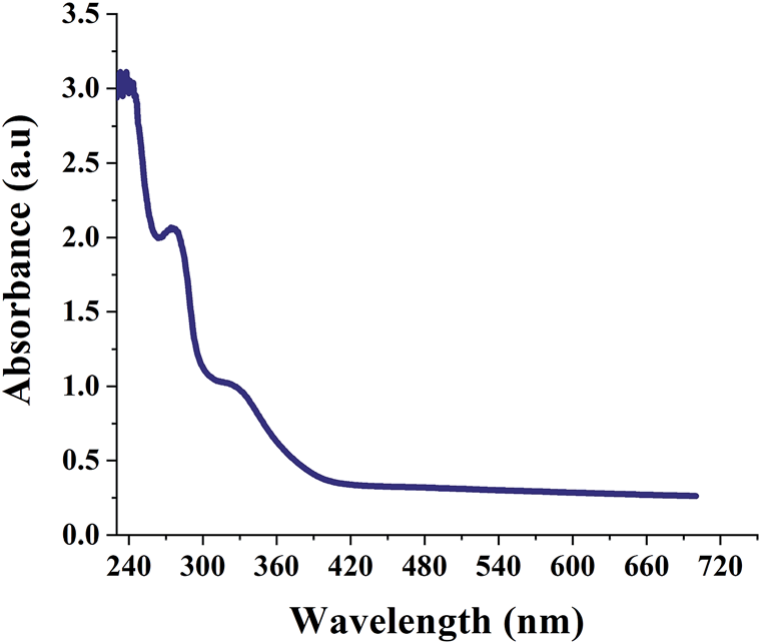
UV-Vis absorption spectrum of biosynthesized SNPs.

Fig. 1 shows the UV-Vis spectrum of SNPs. It is well known that α-sulfur exhibits an optical absorption maximum (*λ*_Max_) in the range of 260–280 nm.^35^ The peak at 274 nm in Fig. 1 indicates the successful formation of SNPs. A secondary peak at ∼324 nm corresponds to the b2 → e3 transition.^36^

**Fig. 2:**
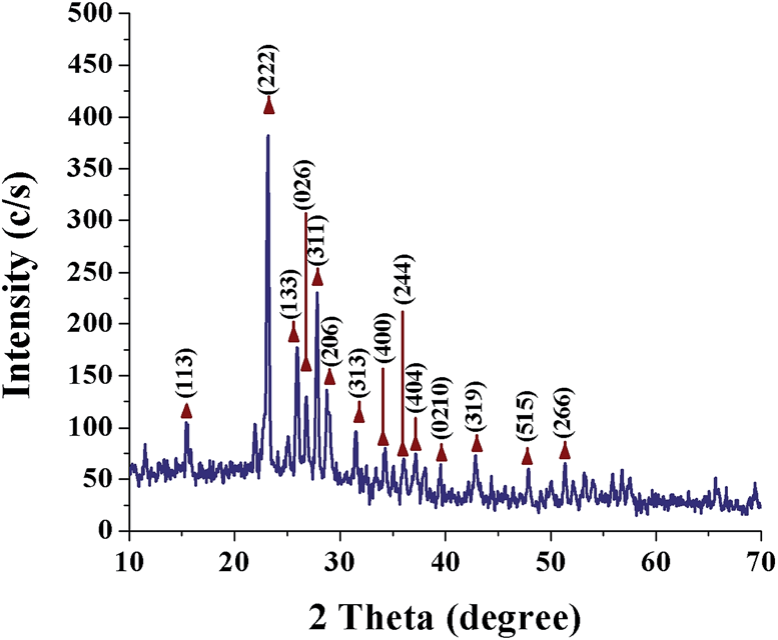
X-ray diffraction pattern of biosynthesized SNPs.

Fig. 2 shows a typical XRD pattern of SNPs. All the diffraction peaks are in good conformity with orthorhombic α-sulfur (JCPDS 000-074-1465) and no other impurity phases are observed, indicating the good phase purity of SNPs. The crystal planes corresponding to the peaks are indicated in Fig. 2. The cell parameters that are calculated from the peak positions are *a* = 10.437 Å, *b* = 12.845 Å, *c* = 24.369 Å. The Debye–Scherrer formula was used to calculate the average crystalline size of the SNPs;

2 *D* = *Kλ*/*β* cos *θ*,

where *β* is the full-width at half maximum, *θ* is the Bragg's diffraction angle, and *K* (=0.9) is the Scherrer constant or the shape factor. Using the diffraction peak associated with the crystal plane (222), the crystallite size was estimated to be 7.2 nm.

**Fig. 3:**
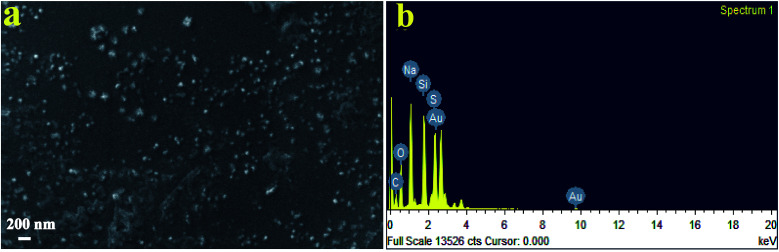
(a) SEM micrograph of biosynthesized SNPs using *F. benghalensis* leaf extract and (b) EDX spectrum of the SNPs.

Fig. 3a shows SEM images of SNPs. It is evident that the particles had a low degree of agglomeration. The SEM micrographs depicted that the particle size ranged from ∼25 to ∼120 nm. The energy dispersive X-ray spectroscopy spectrum (Fig. 3b) showed the presence of sulfur. Other elements such as Na, O and C were also observed due mainly to the presence of the by-product, sodium citrate. Si and Au were appeared due to mica substrates and the sputtered Au, respectively.

**Fig. 4:**
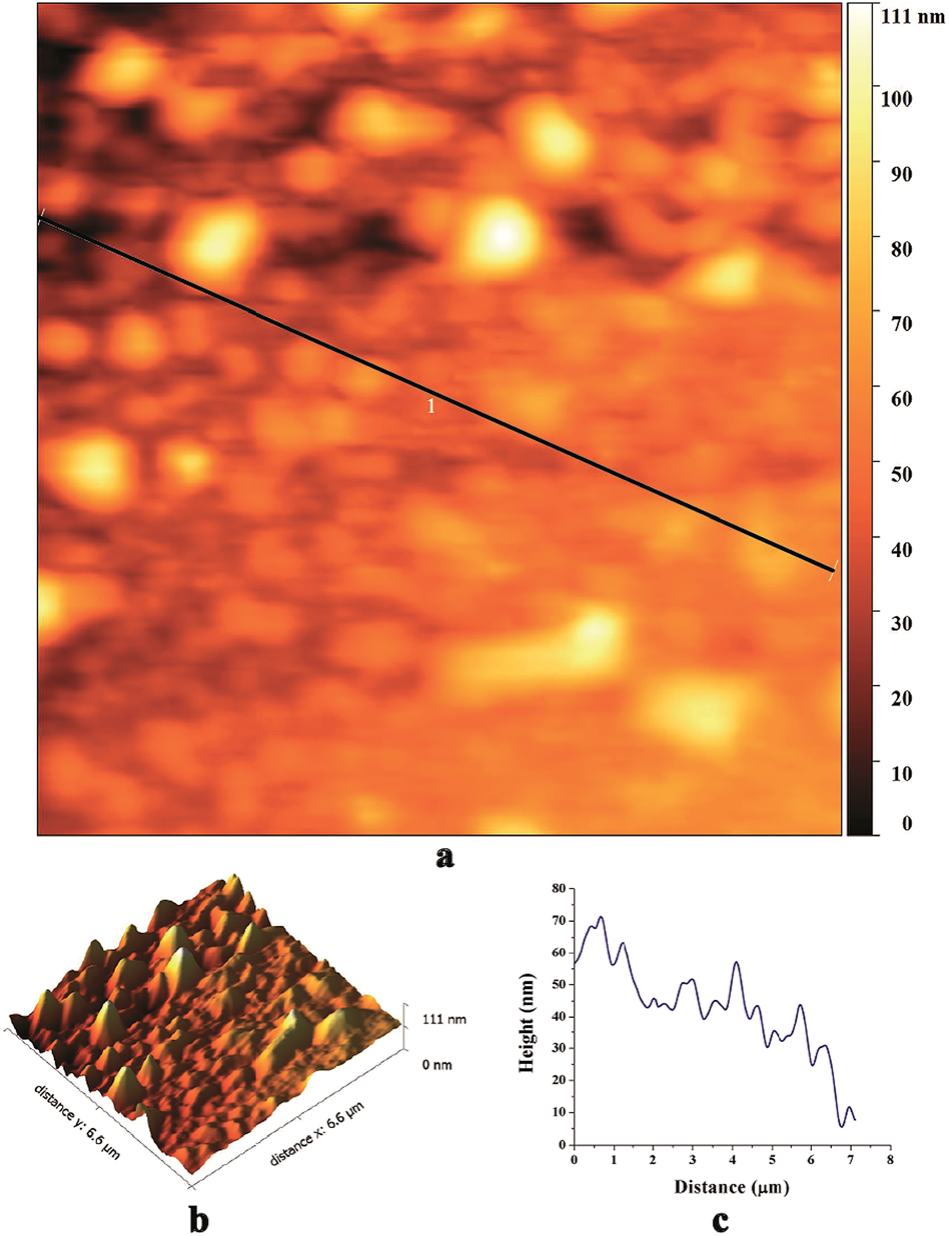
AFM images of the biosynthesized SNPs; (a) a phase diagram, (b) 3D reconstruction and (c) a height curve along the line-1 in the phase diagram.

Fig. 4 shows AFM images of biosynthesized SNPs. The phase diagram (Fig. 4a) shows that small particles are closely anchored throughout the film surface. The height curve along the line in the phase diagram shows the size distribution of SNPs in the range of 10 to 60 nm (Fig. 4c). The average roughness and root mean square roughness values of the SNP layers were 35.1 nm and 41.8 nm, respectively (Fig. 4b).

**Fig. 5:**
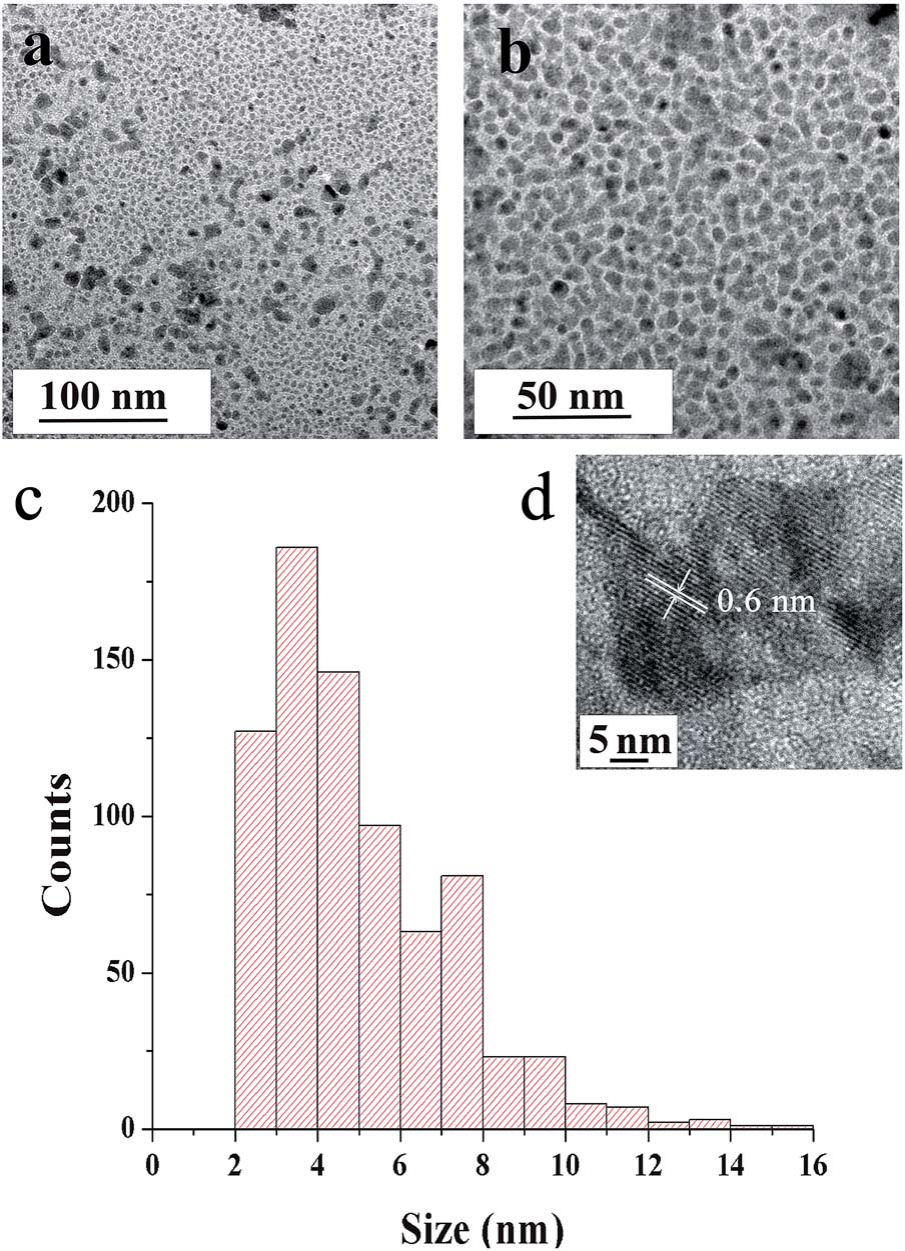
TEM micrographs of SNPs (a) at medium- and (b) high-magnifications and (c) particle size distribution obtained from TEM micrographs (d) high resolution TEM image.

Fig. 5a–c show TEM images of SNPs. The micrographs revel that the primary particles had random shapes with a low degree of agglomeration. The primary particles had a narrow size distribution, with sizes ranging from ∼2 to ∼15 nm and the average size of 5.1 nm (Fig. 5c). The distance between the lattice fringes in Fig. 5d was 0.6 nm, corresponding to (004) crystal plane of orthorhombic sulfur.

**Fig. 6:**
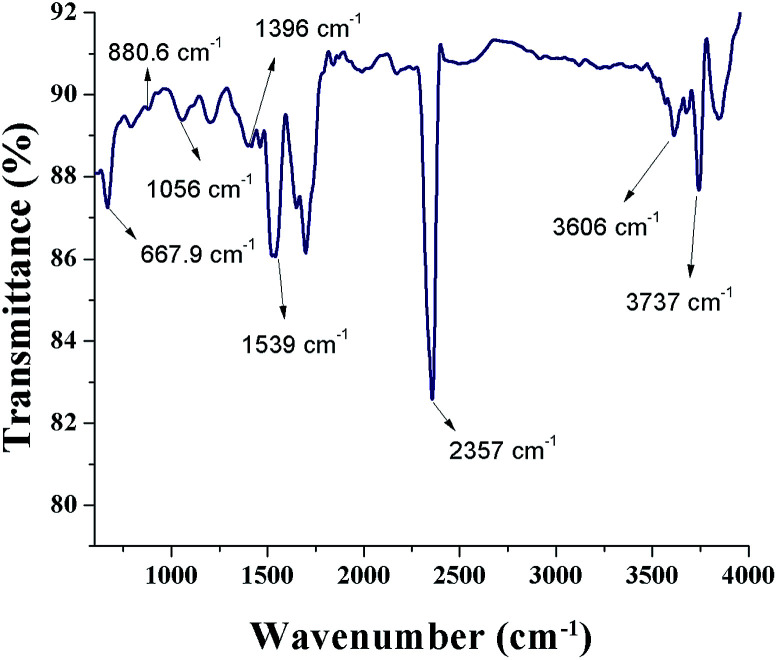
FTIR spectrum of biosynthesized SNPs.

Fig. 6 shows a FTIR spectrum of SNPs. An intense peak at 2357 cm^−1^ corresponds to O=C=O bonds and the peak at 3737 cm^−1^ is due to the external isolated –OH groups.^37^ The symmetric stretching vibration at 1396 cm^−1^ shows the presence of COO^−^ bonds. The peak occurring at 3606 cm^−1^ shows the N–H stretching vibration. The presence of O=C=O bonds, N–H bonds, and COO^−^ bonds indicates that the biomolecules from *F. benghalensis* leaf extract were present in the sample. The peaks at 667.9 cm^−1^, 1056 cm^−1^, 880.6 cm^−1^, and 1539 cm^−1^ correspond to S_8_. The peak positions showed slight shift from the characteristic peaks of S_8_, 656 cm^−1^, 1052 cm^−1^, 876 cm^−1^, 1513 cm^−1^, respectively.^38^ The peak shift arises due to the interaction of SNPs with amine groups in the biomolecules.^26,39^ The results indicate that the biomolecules form the *F. benghalensis* leaf extract proteins were bound onto the surface of as-prepared SNPs.

### Biosynthesis mechanism

**Fig. 7:**
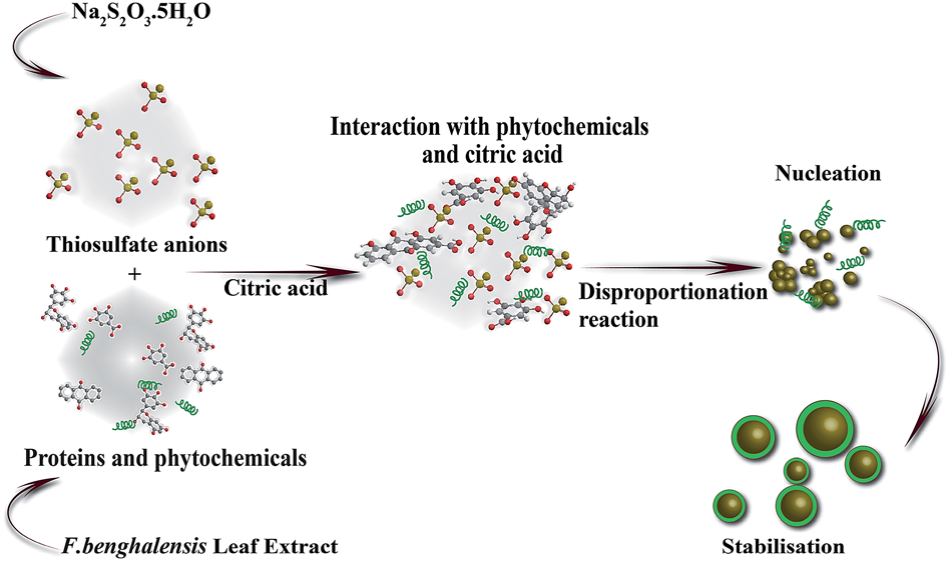
Mechanistic representation of biosynthesis of SNPs from *F. benghalensis* leaf extract.

The possible mechanism of biosynthesis of SNPs is described in Fig. 7. It is well known that the polyphenolic compounds, which exist abundantly in all parts of the plants and trees such as bark, leaves, fruits, seeds, flowers, and pollen, are effective reducing agents to restrain reactive oxygen species (ROS).^40^*F. benghalensis* leaves have a prominent amount of polyphenolic compounds such as gallic acid, rhein, anthraquinone, and gallocatechol.^41^ When these phytochemicals and citric acids are mixed with thiosulfate solutions, instantaneous reduction of S^6+^ to S^0^ by phytochemicals and oxidation of S^2−^ to S^0^ by citric acid occur simultaneously through the disproportionation process. Then S^0^ undergoes a nucleation process to reach its optimum size and shape that are influenced by various parameters such as the nature of reducing and capping agent, temperature, and pH. The presence of O=C=O bonds (2357 cm^−1^), N–H bonds (3606 cm^−1^), and COO^−^ bonds (1396 cm^−1^), in the FTIR spectrum indicates the presence of biomolecules which participated in the synthesis of SNPs.^26^

### Catalytic activity

**Fig. 8:**
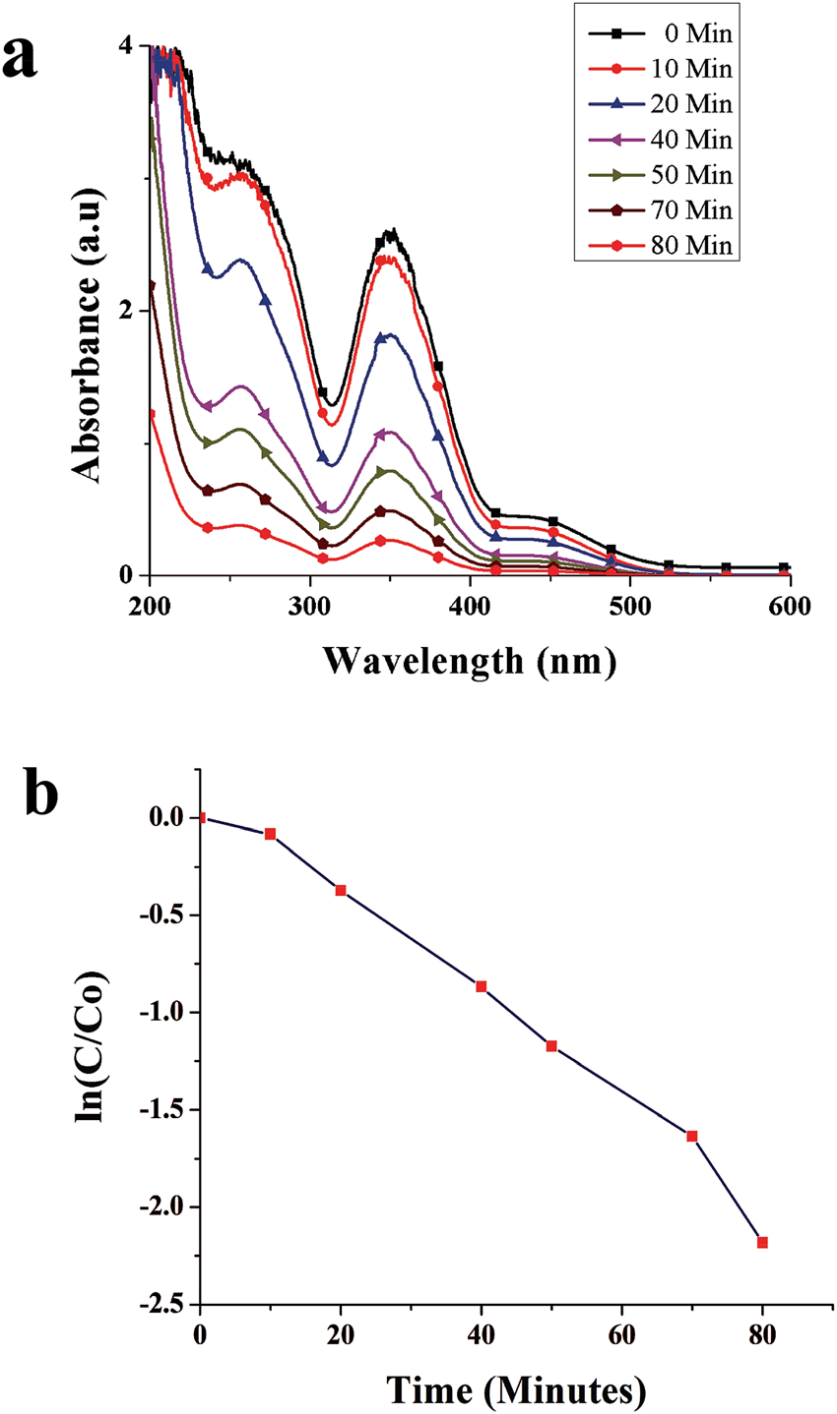
(a) Absorbance spectra of Cr(vi) in the presence of SNPs as a function of time, and (b) catalytic degradation kinetics of Cr(vi).

**Fig. 9:**
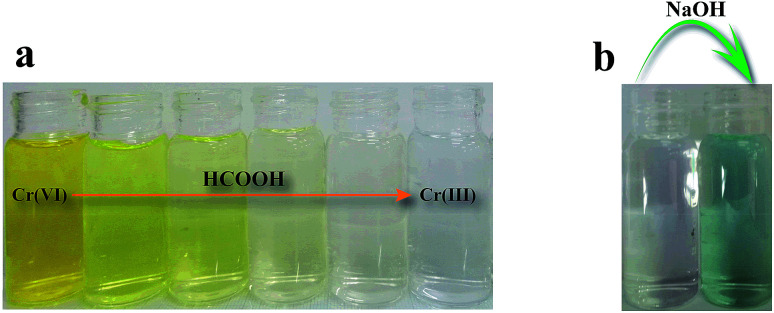
(a) Photograph of the reaction mixtures at various stages of Cr(vi) reduction to Cr(iii), and (b) colorimetric confirmation of the presence of Cr(iii) after catalytic reduction of Cr(vi).

Fig. 8a shows time-dependent absorbance spectra of the Cr(vi) solution at the reaction time from 0 to 80 min. The characteristic absorption peak associated with Cr(vi) decreased as the reaction time increased, which was accompanied by the weakening of the typical yellow color of Cr(vi) in the solution (Fig. 9a), indicating the decrease in Cr(vi) concentration. The conversion of Cr(vi) into Cr(iii) was confirmed by the colorimetric detection of Cr(iii) (Fig. 9b).^34^

The kinetics of Cr(vi) conversion into Cr(iii) was analyzed by calculating ln(*C*/*C*_0_) against time, where *C* represents the concentration of Cr(vi) ion at a specific time and *C*_0_ the initial concentration of Cr(vi). As shown in Fig. 8b, the conversion of Cr(vi) into Cr(iii) showed the first-order kinetics. The rate constant was calculated using the formula

3 ln(*C*/*C*_0_) = −*kt*,

where *t* is the reaction time and *k* is the rate constant. By fitting a straight line onto the data points in Fig. 8b, the rate constant was found to be 0.0268 min^−1^. The conversion rate calculated using eqn (1) was 88.7% in 80 min.

Table 1 compares the performance of selected nanomaterials for the reduction of Cr(vi). Effective turn over frequency (eTOF) was calculated as the product of the first-order reaction rate constant and Cr(vi)/catalyst ratio. As shown in Table 1, the SNPs showed an eTOF higher than that of zero-valent iron nanoparticles. The eTOF was order-of-magnitude higher than the photocatalytic nanoparticles. Although the eTOFs of precious-metal nanocatalysts were significantly higher than that of SNPs, SNPs offers a significant advantage in materials cost.

**Table 1:** Performance of nanomaterials in the reduction of hexavalent chromium

Material	Cr(vi)/catalyst ratio (mol g^−1^)	1^st^ order rate constant (min^−1^)	Effective turnover frequency (mol g^−1^ min^−1^)	Reference
Nanoscale zero-valent sulfur	3.85 × 10^−1^	0.027	1.04 × 10^−2^	Present study
Nanoscale zero-valent iron	6.41 × 10^−4^	0.017	1.09 × 10^−5^	6
Nanoscale zero-valent iron	2.31 × 10^−3^	0.530	1.22 × 10^−3^	42
PtAu NSs/rGO	2.80	0.556	1.56	12
Pd nanowire	8.00	0.282	2.26	43
ZnO nanorods (photocatalytic)	1.92 × 10^−4^	0.016	3.08 × 10^−6^	16
TiO_2_ nanoparticles (photocatalytic)	2.75 × 10^−4^	0.035	9.63 × 10^−6^	44
NiO nanoparticles (photocatalytic)	9.62 × 10^−4^	0.0026	2.50 × 10^−6^	45

**Fig. 10:**
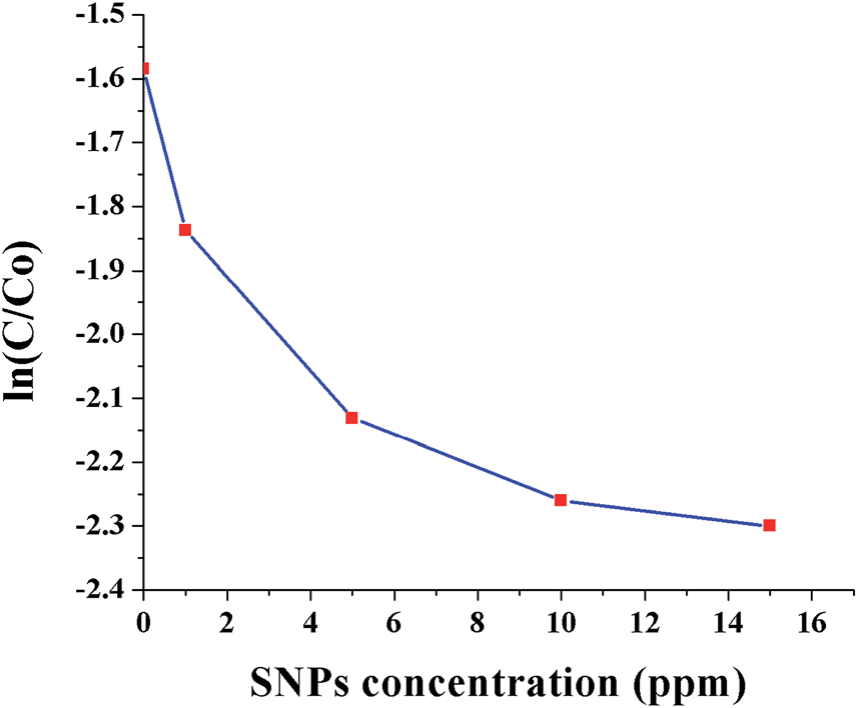
Conversion of Cr(vi) to Cr(iii) as a function of SNPs concentration (0, 1, 5, 10 and 15 ppm).

**Fig. 11:**
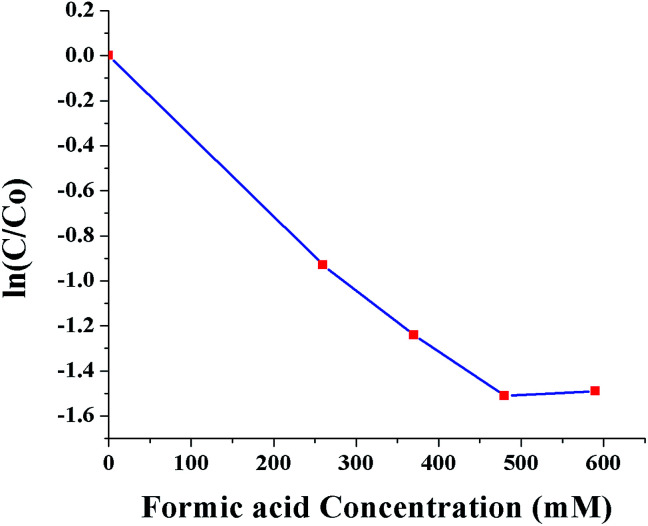
Conversion of Cr(vi) to Cr(iii) as a function of formic acid concentration (0, 260, 370, 480 and 590 mM).

Fig. 10 shows ln(*C*/*C*_0_) as a function of SNPs concentration for the reaction time of 40 min. It was observed that the conversion efficiency increases with increased concentration of SNPs. This was attributed to the greater availability of catalytic sites for Cr(vi) reduction with increased SNPs concentration. When the concentration of SNPs was increased beyond 10 ppm, the conversion efficiency showed little improvement. Fig. 11 shows ln(*C*/*C*_0_) as a function of formic acid concentration for the reaction time of 60 min, in the presence of 10 ppm SNPs. The catalytic reduction of Cr(vi) was increased as the concentration of formic acid increased up to 480 mM and then plateaued. The results indicate that the optimum concentration ratio between SNPs and formic acid is 10 ppm : 480 mM.

**Fig. 12:**
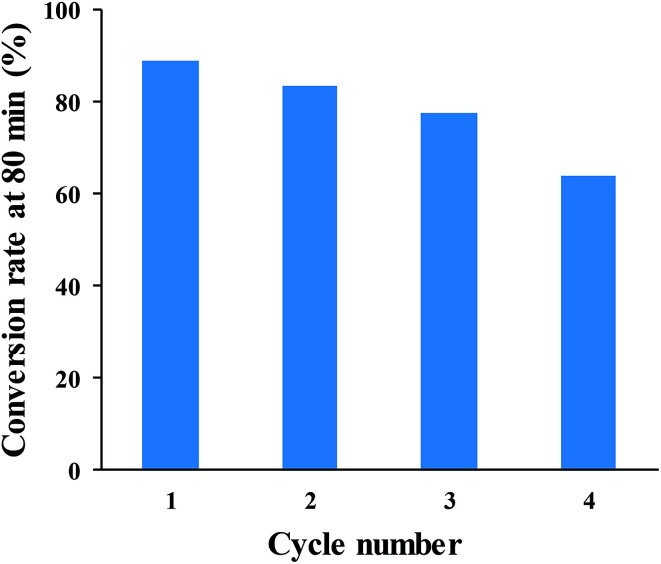
Cr(vi)-to-Cr(iii) conversion rate after 80 min of reaction time, for four successive cycles.

The reusability of SNPs for the catalytic conversion of Cr(vi) was studied. After the completion of the first cycle, SNPs was separated from the solution by centrifuging at 5000 rpm for 10 min and them washed with deionized water. The washed pellet was re-dispersed in the solution of Cr(vi) and HCOOH under the same condition as for the first cycle. The result in Fig. 12 shows that, the conversion rate decreased after each cycle. The initial conversion rate of 88.7% was reduced to 83.5%, 77.3% and 63.9% after 80 min of reaction time, in the consecutive cycles. The decreased rate can be ascribed to particle loss during the recovery process, particle agglomeration, and surface passivation due to the absorption of Cr(iii).

## Conclusions

In the present study, Cr(vi) reduction by zero-valent sulfur nanoparticles as the main catalyst was demonstrated for the first time. A new, cost effective, rapid, environmentally friendly method was used for the synthesis of sulfur nanoparticles using *F. benghalensis* leaf extract. The synthesized nanoparticles had a narrow size range (2–15 nm) and a near-spherical morphology. The FTIR spectra revealed that the protein molecules encapsulated the nanoparticles and provided high dispersion stability. The sulfur nanoparticles was effective in catalytically reducing Cr(vi) into less toxic Cr(iii) in the presence of formic acid. It was found that the optimum concentration ratio between SNPs and formic acid was 10 ppm : 480 mM. Under this condition, the conversion rate of 200 ppm Cr(vi) into Cr(iii) was 88.7% in 80 min of reaction time. The present study provides a strong evidence that, similar to zero-valent iron nanoparticles, zero-valent sulfur nanoparticles have wide-ranging applications in the remediation of heavy metal pollution in water.

## Conflicts of interest

There are no conflicts to declare.

## Supplementary Material
